# Neuroprotective therapies in the NICU in term infants: present and future

**DOI:** 10.1038/s41390-022-02295-2

**Published:** 2022-10-04

**Authors:** Eleanor J. Molloy, Mohamed El-Dib, Sandra E. Juul, Manon Benders, Fernando Gonzalez, Cynthia Bearer, Yvonne W. Wu, Nicola J. Robertson, Tim Hurley, Aoife Branagan, C. Michael Cotten, Sidhartha Tan, Abbot Laptook, Topun Austin, Khorshid Mohammad, Elizabeth Rogers, Karen Luyt, Sonia Bonifacio, Janet S. Soul, Alistair J. Gunn

**Affiliations:** 1 Paediatrics, Trinity College Dublin, Trinity Research in Childhood Centre (TRICC), Dublin, Ireland.; 2 Children’s Hospital Ireland (CHI) at Tallaght, Dublin, Ireland.; 3 Neonatology, CHI at Crumlin, Dublin, Ireland.; 4 Neonatology, Coombe Women’s and Infants University Hospital, Dublin, Ireland.; 5 Department of Pediatric Newborn Medicine, Brigham and Women’s Hospital, Harvard Medical School, Boston, MA, USA.; 6 University of Washington, Seattle, WA, USA.; 7 Department of Neonatology, Wilhelmina Children’s Hospital, University Medical Center Utrecht, Utrecht University, Utrecht, The Netherlands.; 8 Department of Neurology, Division of Child Neurology, University of California San Francisco, San Francisco, CA, USA.; 9 Division of Neonatology, Department of Pediatrics, Rainbow Babies & Children’s Hospital, Cleveland, OH, USA.; 10 Case Western Reserve University School of Medicine, Cleveland, OH, USA.; 11 Department of Neurology, University of California San Francisco, San Francisco, CA, USA.; 12 Institute for Women’s Health, University College London, London, UK.; 13 Centre for Clinical Brain Sciences, University of Edinburgh, Edinburgh, UK.; 14 Department of Pediatrics, Duke University, Durham, NC, USA.; 15 Pediatrics, Division of Neonatology, Children’s Hospital of Michigan, Detroit, MI, USA.; 16 Wayne State University School of Medicine, Detroit, MI 12267, USA.; 17 Pediatrics, Division of Neonatology, Central Michigan University, Mount Pleasant, MI, USA.; 18 Department of Pediatrics, Women and Infants Hospital, Brown University, Providence, RI, USA.; 19 Department of Paediatrics, University of Cambridge, Cambridge, UK.; 20 Section of Neonatology, Department of Pediatrics, University of Calgary, Calgary, AB, Canada.; 21 Department of Pediatrics, University of California, San Francisco Benioff Children’s Hospital, San Francisco, CA, USA.; 22 Translational Health Sciences, University of Bristol, Bristol, UK.; 23 Neonatology, University Hospitals Bristol and Weston NHS Foundation Trust, Bristol, UK.; 24 Division of Neonatal and Developmental Medicine, Department of Pediatrics, Stanford University School of Medicine, 750 Welch Road, Suite 315, Palo Alto, CA 94304, USA.; 25 Department of Neurology, Boston Children’s Hospital, Harvard Medical School, Boston, MA, USA.; 26 Departments of Physiology and Paediatrics, School of Medical Sciences, University of Auckland, Private Bag 92019, Auckland, New Zealand.

## Abstract

Outcomes of neonatal encephalopathy (NE) have improved since the widespread implementation of therapeutic hypothermia (TH) in high-resource settings. While TH for NE in term and near-term infants has proven beneficial, 30–50% of infants with moderate-to-severe NE treated with TH still suffer death or significant impairments. There is therefore a critical need to find additional pharmacological and non-pharmacological interventions that improve the outcomes for these children. There are many potential candidates; however, it is unclear whether these interventions have additional benefits when used with TH. Although primary and delayed (secondary) brain injury starting in the latent phase after HI are major contributors to neurodisability, the very late evolving effects of tertiary brain injury likely require different interventions targeting neurorestoration. Clinical trials of seizure management and neuroprotection bundles are needed, in addition to current trials combining erythropoietin, stem cells, and melatonin with TH.

## INTRODUCTION

**Fig. 1:**
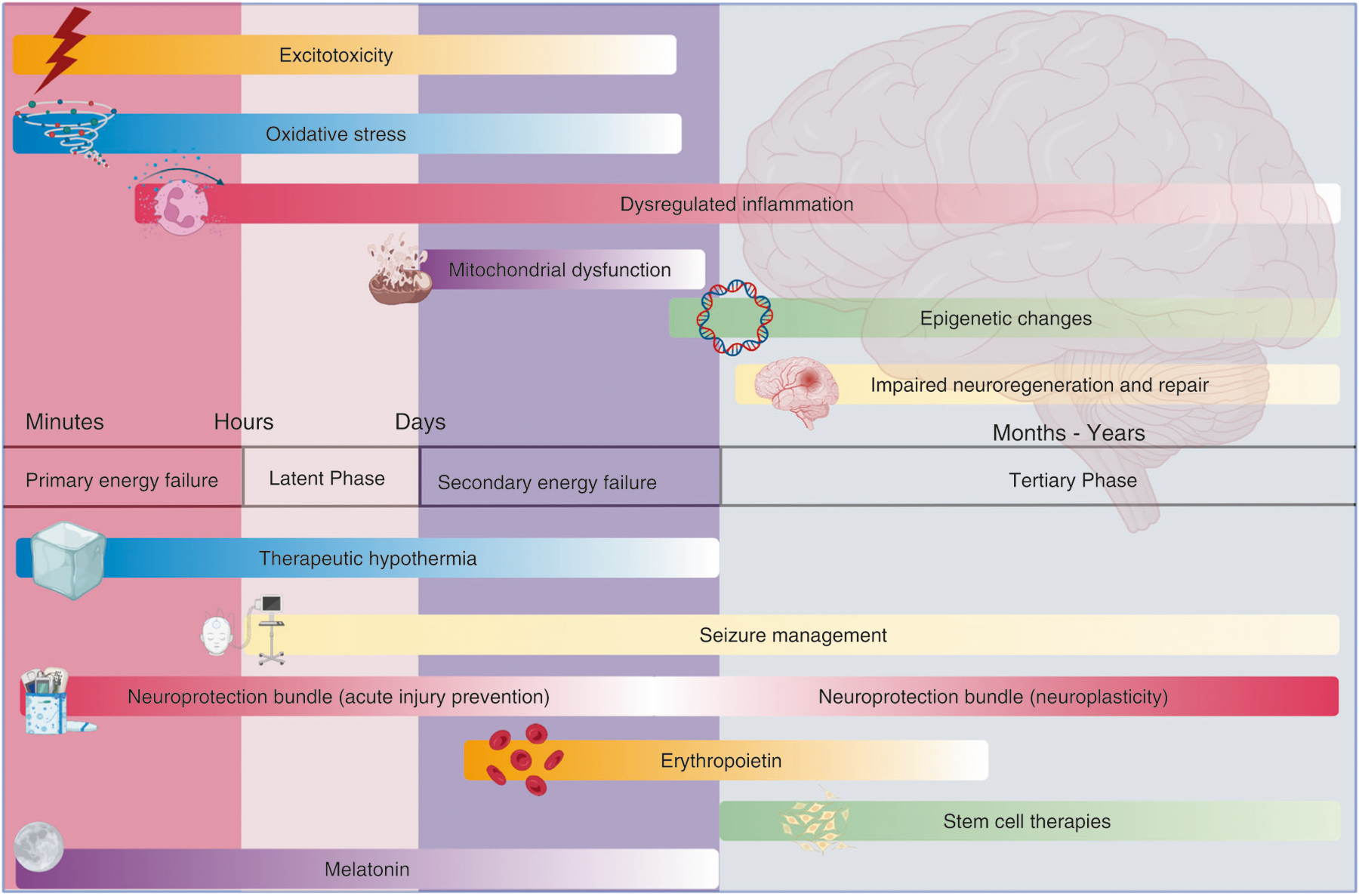
Pathophysiology, phases of injury, and therapeutic windows for present and future neuroprotective interventions in term newborns. Earliest phases of injury are targeted by interventions, including therapeutic hypothermia, acute neuroprotective bundles, and melatonin. Neuroplasticity bundles, erythropoietin, and stem cell therapies aim to reduce injury during the later phases. Improved seizure management offers neuroprotection throughout all stages of injury.

Improvements in antenatal care and advances in neonatal intensive care have reduced neonatal morbidity and mortality. However, adverse neurodevelopmental outcomes remain significant for many children. A variety of complications, such as cerebral congenital malformations, genetic anomalies, congenital epilepsies, and congenital cardiac defects, may also contribute to long-term neurological disability. Neonatal encephalopathy (NE) is a heterogeneous problem that contributes about 700,000 deaths per year worldwide in term and near-term infants^1^ and affects 1–4 per 1000 births in high-resource settings. The development and successful translation of therapeutic hypothermia (TH) has confirmed the fundamental principle that it is possible to reduce the risk of disability after acute hypoxia–ischemia (HI) (Fig. 1).

The seminal finding that underpinned development and translation of TH is that perinatal brain damage after HI evolves over time, with initial transient recovery of oxidative metabolism followed by progressive activation of cell death pathways, leading to secondary deterioration after approximately 4–8 h, with failure of oxidative metabolism, delayed seizures, and ultimately cell death. This delay provides a window of time after HI during which it is possible to intervene with TH. In addition, tertiary mechanisms of brain injury may continue for weeks, months, or years,^2^ involving dysregulated immune responses and loss of trophic support, which may be amenable to novel therapies after the end of hypothermia treatment.^3^

Many different treatment strategies have been postulated as adjunctive treatment strategies to TH to further decrease morbidity. These include allopurinol, azithromycin, ascorbic acid, ibuprofen, magnesium sulfate, xenon gas treatment, and sildenafil. This paper will concentrate on the discussion of melatonin (MT), erythropoietin, and mesenchymal stromal cells as these therapies are undergoing evaluation in human clinical trials at present.

## THERAPEUTIC HYPOTHERMIA

TH is now routine care for infants with moderate-to-severe NE.^4^ It was first been recommended as standard treatment by the International Liaison Committee on Resuscitation (ILCOR) in 2010, based on compelling evidence from randomized controlled trials (RCTs) that TH, and improvements in supportive neonatal intensive care unit (NICU) care during treatment, reduces brain injury detected by modern imaging,^5^ and improves survival and neurological outcomes into middle childhood.^6,7^ The parameters for optimal neuroprotection are now well understood.^8^ Brain temperature needs to be reduced by ~3.5 °C, starting as soon as possible in the first 6 h after HI and then continued for ~72 h. Shorter or longer cooling than 72 h, or deeper cooling (by >5 °C) reduces neuroprotection both in preclinical studies^9–11^ and in a large randomized clinical trial.^12^ Thus, current clinical protocols are close to optimal.

In large randomized trials, hypothermic neuroprotection was incomplete, reducing the combined risk of death and severe disabilities at 18 months of age by ~12%, from 58 to 46%.^13^ Thus, many infants still die or survive with major debilitating handicaps, despite TH intervention. There is evidence that the risk of adverse outcome despite TH has fallen from about 45% in the original trials to about 29% in a recent RCT.^12^ The recent trial of late cooling found an overall risk of adverse outcome of just 26%.^14^ This improvement mainly reflects a reduction in mortality, from 25% in the original trials to 10%, with little change in the rate of disability after NE.

The challenge now is twofold: first, to find ways to improve the outcomes for infants with NE who have been treated with TH, and second, to improve treatment strategies in settings in which TH is not beneficial or is contraindicated, such as low–middle-income countries.^15^ Broadly, the key mechanisms of TH are to attenuate evolving programmed cell death and inflammation, raising the possibility of overlap with the mechanisms of potential adjunct treatment.^16^

## NEUROPROTECTION BUNDLES IN TERM AND NEAR-TERM NEONATES IN NEURONICU

Neonatal brain injury is a complex, multifactorial process.^17^ Genetic, epigenetic, metabolic, prenatal, perinatal, and postnatal factors interact to protect, cause, or exaggerate neonatal brain injury.^18–22^ This complexity makes developing a monotherapy challenging since it is improbable that any one intervention will be applicable in all settings.^23^ There is an increasing interest in a multi-intervention bundled approach using quality improvement methodology to alleviate neonatal brain injury.^24^ Neuroprotection bundles can be divided into (1) acute brain injury prevention and (2) neuroplasticity bundles.

The key concepts in the acute brain injury prevention bundles are early identification and referral, preventing fluctuation in physiologic parameters (such as carbon dioxide, glucose, blood pressure, temperature), minimal handling and pain management, seizure diagnosis and management, early nutrition, and fluid and electrolyte balance.^25–30^ Implementation of neuroprotection bundles targeting those key elements through outreach and Neonatal Neuro-Critical Care programs have proven to be effective in improving NE identification and preventing short term morbidities such as rate of brain injury on magnetic resonance imaging (MRI), antiseizure medication (ASM) doses and timing of treatment, use of boluses and inotropes, temperature fluctuation, and overall hospital length of stay.^31–35^ Evidence for the long-term impact of such programs is still lacking and is required for any of these approaches to become standard care.

Neuroplasticity bundles target potential brain injury and growth well beyond the first few days of birth and after discharge.^36^ Key elements in such bundles (evaluated in preterm and/or term infants) have variably included (1) empowering families through Family Integrated Care (FICARE);^37^ (2) optimizing nutrition;^38^ (3) developmental care;^39^ (4) skin-to-skin care and massage therapy;^40,41^ (5) positive stimulating sounds such as music therapy,^42^ reading programs,^43^ parental voice,^44^ minimizing disturbing noises,^45^ and enhancing physiologic sleep–wake cycles;^46^ and (6) encouraging positive social interaction.^47^ Although there is limited evidence that neuroplasticity interventions can improve long-term cognitive and motor outcomes, well-powered studies are still lacking.^40,41,43^

## SEIZURE MANAGEMENT

One potential neuroprotective strategy is improved treatment of seizures associated with acute neonatal brain injury, i.e., seizures related to hypoxic–ischemic encephalopathy (HIE), stroke, and intracranial hemorrhage (ICH). These three disorders underlie ~75% of neonatal seizures.^48–50^ With increasing recognition of the association between neonatal seizures and adverse outcomes, there has been increased attention and research effort on the improved detection and management of acute symptomatic neonatal seizures using gold standard continuous, conventional video electroencephalograph (EEG) monitoring.^51–53^ Similarly, there has been increased interest in developing and testing more effective and safer treatments for neonatal seizures.^54^ The direction of causality is still not clear.

TH reduces seizure burden substantially in cohort studies of infants with moderate-to-severe HIE compared to normothermia.^55–57^ Interestingly, some studies found this only after moderate HIE, whereas others report an improvement with severe HIE as well.^55^ The overall incidence of seizures is reported not to be affected by TH, suggesting that the duration of individual seizures and the total time of seizures is lower in infants who do seize,^57^ consistent with animal studies.^58^

Experimental models have shown that intense neonatal seizures by themselves can lead to decreased neurogenesis, synaptic reorganization, dendritic spine loss, and other effects on the developing brain that correlate well with later cognitive deficits, such as memory impairments.^59^ However, it is challenging to disentangle the impact of seizures from brain injury in experimental models of HI injury without seizures. For example, injection of the excitotoxin kainic acid in normoxic P10 rat induced clinical and electrographic seizures lasting a mean of 282 min, but notably did not cause brain injury after either 3 or 20 days recovery.^60^ The authors then tested the effect of seizures after a model of HI for 30 min that induced moderate neuropathological injury, but no electrographic seizures. Kainic acid injection after this period of HI induced superimposed seizures and increased neuronal loss in the hippocampus. Critically, a subsequent study found that the kainite-induced seizures were associated with a small increase in brain temperature—and that preventing hyperthermia abolished the increase in neuronal necrosis up to 20 days of recovery.^61^ Thus, spontaneous seizures may not exacerbate injury after HI, and at least part of their injurious effects may be mediated by hyperthermia, consistent with association of pyrexia in multiple preclinical studies and clinical trials of infants with neonatal encephalopathy.^62,63^ Conversely, in near-term fetal sheep receiving cerebral ischemia that led to status epilepticus and severe watershed brain injury, treatment with the potent anti-excitotoxic agent dizocilpine starting at 6 h, before the onset of seizures, completely abolished seizures but had only a modest effect to reduce injury in mildly affected regions and no effect on parasagittal cortical infarction.^64^

Determining the direction of causality in humans is challenging, as it is impossible to determine whether more severe brain injury begets more severe seizures, or the reverse, or a combination of both. There are data showing that higher seizure burden is associated with worse short- and long-term outcomes,^50,65^ although the higher seizure burden may reflect greater injury. One small study suggested that seizure severity was associated with outcome independently of severity of HI injury by MRI.^66^ Two small, randomized trials of treatment of clinical vs EEG-proven seizures also suggested that higher seizure burden is associated with worse brain injury and neurodevelopmental outcome.^67,68^ Although these trials were small, treatment of EEG-proven seizures resulted in reduced seizure burden compared with treatment of only clinical seizures, showing that EEG-guided treatment is more effective in reducing seizure burden than treatment of only clinically suspected seizures. Notably, both trials found that the reduced seizure burden was associated with less brain injury by brain MRI and improved neurologic outcome. Although these data suggest that higher seizure burden is harmful, the clinical impact of mild-to-moderate seizure burden is unclear. An expert consensus recommended a threshold of 30 s/h of seizure activity for randomization in a clinic trial,^69^ but the threshold that should prompt treatment in routine clinical care is unknown, and requires further research.

Importantly, the ASMs used to treat neonatal seizures are often ineffective, as ~40–60% of neonates will have persistent seizures after an initial loading dose of an ASM.^50,70^ Moreover, there is limited evidence for the efficacy and safety of ASMs for neonatal seizures,^71^ as there have been only three randomized trials of ASMs to treat neonatal seizures,^70,72,73^ two of which used a crossover design,^70,73^ in addition to open-label, uncontrolled studies.^74–77^ Phenobarbital and phenytoin had efficacy in the first RCT in which EEG monitoring was not continuous.^70^ More recently, levetiracetam 40–60 mg/kg was shown to be much less effective than phenobarbital 20–40 mg/kg, albeit with a marginally better adverse effect profile.^73^ Add-on treatment with bumetanide enhanced seizure reduction in a randomized, double-blind controlled trial, particularly with higher bumetanide exposure, compared with standard therapy alone (phenobarbital).^72^ This promising result will need to be tested in a larger trial to determine ideal dose, efficacy, and safety before it is incorporated into clinical care, as this class of drugs can be ototoxic. Other ASMs such as lidocaine and midazolam have been studied only in small cohort and/or retrospective studies and had low reported efficacy.^74,76^ Some of the ASMs used widely in older children, such as topiramate, which could have an additional neuroprotective effect, have been rarely used or studied.^78,79^ In a randomized trial of 110 infants with HIE, add-on therapy with topiramate (by nasogastric tube) vs placebo with TH (HYPOTOP), topiramate reduced seizures in the subset who reached therapeutic levels in the first 24 h but had no significant effect on mortality or neurodevelopmental outcomes at 2 years.^80^ The lack of adequate efficacy and safety data for currently used ASMs speaks of the compelling need to develop novel ASMs targeted to the specific pathophysiology of neonatal seizures and to test their efficacy and safety in rigorously designed RCTs that balance important covariates such as severity of both neonatal encephalopathy and seizures.^69^

Animal models have raised concerns about the potential effects of frequently used ASMs especially in the area of brain development and neurodevelopmental outcome. Phenobarbital was seen to induce apoptosis in rodent neurons in the cortex, hypothalamus, thalamus, basal ganglia, and the developing white matter, however, at a higher dose than typically used clinically.^81–83^ In rats, a threshold dose of 40 mg/kg was associated with apoptosis. Importantly, when phenobarbital was combined with diazepam, even lower doses were associated with apoptosis.^83^ Phenobarbital and phenytoin have been shown to disrupt the maturation of synapses in the developing brain of the neonatal rate and thus impair behavior.^84^ In comparison, although levetiracetam seems to be a less effective anticonvulsant it has a superior safety profile,^85^ and there is some evidence from rat models that it may reduce apoptosis after HI.^86,87^

## ERYTHROPOIETIN IN TERM INFANTS

Erythropoietin (Epo) is a 30.4-kDa glycoprotein primarily produced in the liver in the fetus and in the kidney and liver after the neonatal period. Epo and its receptor (Epo-R) are expressed by many cell types in the brain. In animal models, Epo can modulate inflammation, angiogenesis, and neurogenesis and promotes white matter development.^88,89^ The response to injury is mediated via hypoxia-inducible factor-1-mediated increase in Epo expression, signaling protein Janus kinase 2, and downstream phosphatidylinositol 3-kinase/Akt, Stat5, and the extracellular signal-regulated kinase.

Serum Epo levels are significantly elevated in both term and preterm infants with adverse neurodevelopmental outcomes and remain dysregulated in later childhood post-NE.^3,90–93^ Recombinant Epo may upregulate Epo receptors in animal models of neonatal HI and Epo levels for tissue protection may be up to 1000-fold higher than required for erythropoiesis.^94^

Numerous studies of Epo neuroprotection performed in rodents, sheep, and nonhuman primates have provided consistent evidence that exogenous Epo results in both histologic and functional benefit, with most benefit seen in multiple, high-dose treatment regimens.^95^ There is a U-shaped dose–response curve, with extremely high doses resulting in a loss of neuroprotection or even increased vulnerability.^96,97^ Less than 1% of circulating Epo crosses the intact blood–brain barrier (BBB), most likely via passive diffusion.^98^ In contrast, higher doses of Epo have been shown in rats, primates, and humans to achieve significant elevations in CSF Epo concentrations, particularly after HI when permeability of the BBB is increased.^99^ Of concern, recent large animal studies found that combined therapy using continuous Epo infusion with TH after HI does not seem to be additive.^100,101^

In humans, Epo monotherapy for neonatal encephalopathy has been tested in small clinical trials in settings where TH was not available.^102,103^ These studies suggest short-term neurodevelopmental benefit after high-dose Epo over the first 5 days of life or three times per week for 2–4 weeks, or long-term benefit with every other day dosing for 2 weeks. No safety concerns have been reported, but their small sample sizes limit extrapolation of results for clinical use of Epo for presumed NE.

In hospitals where TH is standard of care, Epo must be studied in this context to examine both safety and long-term efficacy. Phase I and phase II trials of combination Epo and hypothermia therapy have demonstrated safety of high-dose Epo, with a dose of 1000 U/kg intravenously (IV) achieving serum concentrations that most closely approximated optimal neuroprotective levels in preclinical models.^96^ The phase II NEATO trial EPO boluses at 1, 2, 3, 5, and 7 days of age may provide additional benefit in MRI injury scores and motor outcomes at 12 months of age.^104^ However, two children in the standard care group had confounding conditions; if these infants are excluded, there was no significant difference between groups. Combination therapy was also found to reduce serum tau protein but did not affect neurodevelopmental outcome at 9 months of age.^105^

Unfortunately, consistent with this interpretation, the phase III High-Dose Erythropoietin for Asphyxia and Encephalopathy Trial (HEAL, NCT# 02811263) that randomized infants to either 1000 U/kg of Epo (*n* = 257) or saline placebo (*n* = 243) given IV within 26 h after birth, and then at 2, 3, 4, and 7 days,^106^ found no effect on the risk of death or neurodevelopmental impairment at 22–36 months of age (52.5% after Epo vs 49.5% after placebo). Moreover, Epo was associated with a higher rate of serious adverse events. The similar, phase III, Preventing Adverse Outcomes of Neonatal Hypoxic Ischemic Encephalopathy with Erythropoietin (PAEAN) Trial (NCT# 03079167) is in progress.^107^

Darbepoetin, a long-acting erythropoiesis-stimulating agent that may provide similar neuroprotective benefit as Epo with a more practical dosing schedule, is also currently being investigated in cooled neonates with NE (DANCE trial: NCT01471015)^108^ and as monotherapy for milder NE (MEND Trial: NCT03071861).^109^ No trials have directly compared these agents.

## STEM CELL THERAPIES

Volume and red blood cell reduced human umbilical cord blood mononuclear cells (hUCB cells), collected and processed with established procedures, have been used for allogeneic transplants, for hematopoietic disorders, as well as inherited metabolic disease^110^ (https://www.fda.gov/home). Mesenchymal stromal cells (MSCs), which have been found in multiple tissues and have been phenotypically defined in a standardized way, have been tested in hundreds of clinical trials, including studies that enrolled hundreds of children testing MSCs as potential therapy for graft vs host disease.^111–115^ Meta-analysis of 46 trials of MSCs in a wide variety of species, including humans, rats, and mice, and in adult animals with stroke indicate improved outcome with MSC treatment compared with placebo.^116^ There was no apparent effect of the origin of the MSCs or the target species, administration route, timing, immunogenicity, or dose.

In neonatal animal studies, both hUCB cells and MSCs have shown promise for neuroprotection after HI.^117–122^ In small and large animal studies, treatment after HI with hUCB cells increases neurotrophic and angiogenic factors, decreases inflammation and microglial activation, and modifies T lymphocyte migration into injured areas of the brain. MSCs work mainly via paracrine secretion of multiple cytokines, morphogens, small molecules, and exosomes, which carry a variety of substances, which can affect the biology of adjacent and distant responder cells and tissue.^112^ Recent in vitro studies describe the formation of membranous channels between MSCs and injured cells (tunneling nanotubules (TNT)); MSCs are thought to inhibit apoptosis and restore cellular bioenergetics by transferring healthy mitochondria to injured cells through TNT.^123^ Exosomes from MSCs may also promote regenerative responses from the neurogenic stem cell niche.^124^ In addition to decreasing markers of inflammation, administration of MSCs in a neonatal brain injury model was associated with increased differentiation towards neurons and oligodendrocytes and decreased proliferating inflammatory cells post-injury. Repeat dosing, several days after injury, seemed to enhance cell differentiation and functional outcome.^122^ Although preclinical trials where MSCs were administered before and immediately after TH were associated with improved anatomic and functional outcomes, one study in P9 mice showed that administering MSCs 3 days after TH for 4 h might be deleterious.^125^ In neonatal piglets, cooling from 1 to 13 h after HI plus intranasally administered MSCs at 24 and 48 h was associated with (i) faster aEEG recovery after injury; (ii) improved brain energy metabolism based on phosphorus-31 magnetic resonance spectroscopy (MRS) but not the Lac/NAA ratio; (iii) reduced total number of TUNEL-positive cells and increased oligodendrocytes in the white matter compared to cooling alone,^126^ but had no effect on gray matter. It is unknown whether these limited benefits would be achieved after a full clinical protocol of TH. In addition to inherent properties of MSCs and their exosomes, further benefits may be achieved by modifications to enhance production of certain neurotrophic factors.^115^ One key issue is that the immunomodulatory effects of MSCs appear to be determined by local inflammatory conditions in the host, with polarization of MSCs to pro-inflammatory or anti-inflammatory phenotype depending on the initial inflammatory milieu.^127^ Therefore, the timing of MSCs administration may be critical in determining the therapeutic response.

Human trials for the use of hUCB cells and MSCs are still at an extremely early stage. Two studies in human infants with moderate-to-severe NE have been published, demonstrating safety and feasibility of collection and preparation of the nucleated cord blood cells.^128,129^ In addition, a small phase I/II, open-label, single-arm study, which evaluated the safety and tolerability of intranasally administered MSC for perinatal arterial ischemic stroke (Perinatal Arterial Stroke Treated With Stromal Cells Intranasally, PASSIoN), has just completed enrolment (NCT03356821: https://clinicaltrials.gov/ct2/show/NCT03356821). The most promising study was a phase II multi-site double-blinded RCT, which aimed at assessing the neuroprotective efficacy of autologous hUCB cells in neonates with moderate-to-severe NE. That study was stopped prematurely after randomization of 35 out of the planned 160 infants due to slow enrolment and funding difficulties (NCT02612155: https://clinicaltrials.gov/ct2/show/NCT02612155). While short-term safety signals have been reassuring, much work is needed to establish the safety and efficacy of cell therapy for brain injury in newborn infants.

## MELATONIN

MT is an endogenous hormone released by the pineal gland. Its release is inhibited by light stimulation so there is significant 24-h variation in MT levels, with higher nighttime and lower daytime physiological concentration.^130^ MT easily crosses the BBB^131^ and MT receptors are widely distributed in different brain regions^132^ and among a wide variety of immune cells, including neutrophils, monocytes, and microglia.^133^ MT has anti-inflammatory properties primarily through prevention of inflammasome activation^134^ and inhibition of pro-inflammatory cytokines production. It also has antioxidant properties as a direct free radical scavenger and by upregulating antioxidant enzymes through activation of MT receptors MT1 and MT2,^135^ and anti-apoptotic properties by preventing mitochondrial release of cytochrome C and apoptosis-inducing factor.^136^ MT is a chronobiotic agent that regulates other circadian rhythms including the expression of circadian rhythm genes,^137^ which exert a major influence on inflammatory responses and immune function.^138^

Evidence from several animal studies including piglets, lambs, sheep, and rats have demonstrated compelling neuroprotective benefits of MT as a single therapy and as an adjunct therapy with TH. In a piglet model of perinatal asphyxia, piglets that received TH plus 30 mg/kg MT within 10 min of HI had improved markers of neuronal viability on MRS and reduced markers of neuronal cell death compared to those that received TH alone.^139^ Further studies in piglets and fetal sheep suggest that the benefit of MT appears to be time critical, dependent on therapeutic levels (15–30 mg/L) achieved within 3–4 h after HI, and that formulations with ethanol excipient are most effective.^140–143^

More recently, small pilot studies in human neonates suggest possible neuroprotective benefits of MT as an adjunctive therapy to TH. The first small RCT of 25 infants with NE to examine the long-term effects of MT (5 mg/kg IV) as an adjunct to TH found that patients receiving MT treatment had better cognitive ability on Bayley-III at 18 months of age compared to those receiving placebo. There was no difference in survival or incidence of cerebral palsy between groups. However, the trial was not powered to detect a difference.^144^ An earlier small trial also found improved survival with reduced neurodevelopmental abnormalities at 6 months of age in patients who received MT (5 daily enteral doses of 10 mg/kg) and TH compared to TH alone.^145^ Two further studies compared MT monotherapy orally to placebo and reported reduced mortality,^146,147^ using eight 10 mg/kg doses every 2 h and a one-off dose of 10 mg/kg, respectively. A recent systematic review and meta-analysis described the paucity of high quality RCTs of MT as an adjunct to TH in NE due to inadequate sample size, subtherapeutic levels with uncertain oral bioavailability in sick neonates, no pharmacokinetic studies and no consistent validated outcome measure.^148^ Large clinical trials of MT are needed.

The Use of Melatonin for Neuroprotection in Asphyxiated Newborns (MELPRO) study, the first phase III placebo-controlled trial of enteral MT as an adjunctive therapy to TH, is currently recruiting and will report on the primary outcome of Bayley scale III neurodevelopmental outcome at 12 months and secondary outcomes of neurodevelopmental outcomes at 24 months, MRI, and aEEG results (NCT03806816). One hundred neonates with moderate-to-severe NE will be randomized to TH or TH with 5 daily enteral doses of MT 10 mg/kg. The bioavailability of enteral MT may be variable in sick neonates undergoing TH; however, serum MT and autophagy levels will be measured at enrolment, daily during TH, at days 5 and 7.

Other proposed interventions include the nonpsychotropic cannabinoid, cannabidiol (CBD), and allopurinol. CBD has been safely used in the treatment of seizures resistant to other ASMs in the pediatric population.^149^ CBD has shown mixed results after HI, with some short-term evidence of benefit after immediate IV infusion after HI in piglets during normothermia or TH.^150,151^ However, other studies found no evidence of neuroprotection, and hypotension developed during higher-dose therapy.^152,153^ Thus, further preclinical studies are needed resolve its potential value, and practical constraints before it can be considered for translation. Allopurinol, a xanthine-oxidase inhibitor, is currently being assessed in a multicenter RCT in 13 European centers as an adjunctive treatment to TH.^154^

## CONCLUSIONS

Following TH, a significant proportion of neonates with NE still develop long-term neurodisability. Therefore, optimizing and further improving neonatal intensive care and neonatal neurocritical care is vital. Additional neuroprotective interventions such as erythropoietin, MT, and stem cells are currently being tested in clinical trials. Follow-up from hospital discharge through childhood to optimize systemic and neurodevelopmental outcomes will be valuable as they may be opportunities for further neuroprotective therapies to prevent tertiary brain injury. The recently established Newborn Brain Society (newbornbrainsociety.org) will have an important role in setting clinical practice guidelines for caring of these infants, facilitating international registries, and organizing/coordinating multicenter research activities to advance this important field.

## Data Availability

Data sharing is not applicable to this article as no datasets were generated or analyzed during the current study.

## IMPACT:

- The widespread use of therapeutic hypothermia (TH) in the treatment of neonatal encephalopathy (NE) has reduced the associated morbidity and mortality. However, 30–50% of infants with moderate-to-severe NE treated with TH still suffer death or significant impairments.
- This review details the pathophysiology of NE along with the evidence for the use of TH and other beneficial neuroprotective strategies used in term infants.
- We also discuss treatment strategies undergoing evaluation at present as potential adjuvant treatments to TH in NE.
